# Trade-offs between risks of predation and starvation in larvae make the shelf break an optimal spawning location for Atlantic bluefin tuna

**DOI:** 10.1093/plankt/fbab041

**Published:** 2021-06-22

**Authors:** Taylor A Shropshire, Steven L Morey, Eric P Chassignet, Mandy Karnauskas, Victoria J Coles, Estrella Malca, Raúl Laiz-Carrión, Øyvind Fiksen, Patricia Reglero, Akihiro Shiroza, José M Quintanilla Hervas, Trika Gerard, John T Lamkin, Michael R Stukel

**Affiliations:** EARTH, OCEAN AND ATMOSPHERIC SCIENCE, FLORIDA STATE UNIVERSITY, TALLAHASSEE, FL 32306, USA; CENTER FOR OCEAN-ATMOSPHERIC PREDICTION STUDIES, FLORIDA STATE UNIVERSITY, TALLAHASSEE, FL 32306, USA; DIVISION OF COASTAL SCIENCES, UNIVERSITY OF SOUTHERN MISSISSIPPI, HATTIESBURG, MS 39406, USA; CENTER FOR OCEAN-ATMOSPHERIC PREDICTION STUDIES, FLORIDA STATE UNIVERSITY, TALLAHASSEE, FL 32306, USA; SCHOOL OF THE ENVIRONMENT, FLORIDA A&M UNIVERSITY, TALLAHASSEE, FL, 32307, USA; EARTH, OCEAN AND ATMOSPHERIC SCIENCE, FLORIDA STATE UNIVERSITY, TALLAHASSEE, FL 32306, USA; CENTER FOR OCEAN-ATMOSPHERIC PREDICTION STUDIES, FLORIDA STATE UNIVERSITY, TALLAHASSEE, FL 32306, USA; SOUTHEAST FISHEIRES SCIENCE CENTER, NATIONAL MARINE FISHERIES SERVICE, MIAMI, FL, 33149, USA; HORN POINT LABORATORY, UNIVERSITY OF MARYLAND CENTER FOR ENVIRONMENTAL SCIENCE, CAMBRIDGE, MD, 21613, USA; SOUTHEAST FISHEIRES SCIENCE CENTER, NATIONAL MARINE FISHERIES SERVICE, MIAMI, FL, 33149, USA; COORPERATIVE INSTITUTE FOR MARINE AND ATMOSPHERIC STUDIES, UNIVERSITY OF MIAMI, MIAMI, FL, 33149, USA; CENTRO OCEANOGRáFICO DE MáLAGA, INSTITUTO ESPAñOL DE OCEANOGRAFíA, FUENGIROLA, Spain; DEPARTMENT OF BIOLOGICAL SCIENCES, UNIVERSITY OF BERGEN, BERGEN, Norway; CENTRE OCEANOGRáFIC DE LES BALEARS, INSTITUTO ESPAñOL DE OCEANOGRAFíA, PALMA DE MALLORCA, Spain; SOUTHEAST FISHEIRES SCIENCE CENTER, NATIONAL MARINE FISHERIES SERVICE, MIAMI, FL, 33149, USA; COORPERATIVE INSTITUTE FOR MARINE AND ATMOSPHERIC STUDIES, UNIVERSITY OF MIAMI, MIAMI, FL, 33149, USA; CENTRO OCEANOGRáFICO DE MáLAGA, INSTITUTO ESPAñOL DE OCEANOGRAFíA, FUENGIROLA, Spain; SOUTHEAST FISHEIRES SCIENCE CENTER, NATIONAL MARINE FISHERIES SERVICE, MIAMI, FL, 33149, USA; SOUTHEAST FISHEIRES SCIENCE CENTER, NATIONAL MARINE FISHERIES SERVICE, MIAMI, FL, 33149, USA; EARTH, OCEAN AND ATMOSPHERIC SCIENCE, FLORIDA STATE UNIVERSITY, TALLAHASSEE, FL 32306, USA; CENTER FOR OCEAN-ATMOSPHERIC PREDICTION STUDIES, FLORIDA STATE UNIVERSITY, TALLAHASSEE, FL 32306, USA

**Keywords:** larval mortality, starvation, predation, individual-based model, physical–biogoechemical model, critical period, Atlantic bluefin tuna, *Thunnus thynnus*

## Abstract

Atlantic bluefin tuna (ABT) (*Thunnus thynnus*) travel long distances to spawn in oligotrophic regions of the Gulf of Mexico (GoM) which suggests these regions offer some unique benefit to offspring survival. To better understand how larval survival varies within the GoM a spatially explicit, Lagrangian, individual-based model was developed that simulates dispersal and mortality of ABT early life stages within realistic predator and prey fields during the spawning periods from 1993 to 2012. The model estimates that starvation is the largest cumulative source of mortality associated with an early critical period. However, elevated predation on older larvae is identified as the main factor limiting survival to late postflexion. As a result, first-feeding larvae have higher survival on the shelf where food is abundant, whereas older larvae have higher survival in the open ocean with fewer predators, making the shelf break an optimal spawning area. The modeling framework developed in this study explicitly simulates both physical and biological factors that impact larval survival and hence could be used to support ecosystem based management efforts for ABT under current and future climate conditions.

## INTRODUCTION

Broadcast spawning fish species, such as Atlantic bluefin tuna (ABT) (*Thunnus thynnus*), typically produce enormous numbers of eggs that experience exceedingly high levels of mortality upon hatching and throughout the early life stages. Mortality during these stages is a result of three main sources: predation, starvation and losses due to advection (e.g. individuals transported away from habitat needed for settlement). Predation is often considered the largest source of mortality because it occurs during all early life stages (Peck and Hufnagl, 2012). However, depending on the species and its habitat, the magnitude of these mortality sources may vary substantially. For example, advective losses are thought to be a significant source of mortality for coastal demersal species whose larvae require specific benthic substrates for settlement (Bailey, 1981). In contrast, starvation is hypothesized to be an important source of larval mortality for oceanic species that spawn in warm oligotrophic seas (Young and Davis, 1990).

Quantifying mortality of early life stages in the field is exceedingly difficult, hence, spatially explicit individual-based models (IBMs) provide a useful strategy for investigating the relationships between mortality and environmental conditions. Many studies have utilized ocean models to investigate larval mortality. Early spatially explicit larval IBMs were used to investigate advection-based losses (Hinckley *et al.*, 1996; Werner *et al.*, 1996; Heath and Gallego, 1998; Hinrichsen *et al.*, 2002). Later studies included foraging, bioenergetics and larval behavior to add further realism and identify key factors that contribute to larval mortality (Daewel *et al.*, 2008; Fiksen and Jørgensen, 2011; Staaterman and Paris, 2014). Despite these advances, modeling larval growth and mortality is often restricted to statistical relationships with temperature, idealized prey fields or static prey fields due to limited observations for characterizing dynamic zooplankton fields. Including realistic larval prey fields is particularly relevant for estimating mortality in species like ABT which spawn in tropical regions where prey availability may be more determinant than temperature for limiting larval growth (Jenkins *et al.*, 1991; Tanaka *et al.*, 2006).

Due to its high economic value and history of overfishing, ABT ecology has been studied extensively, including the early life stages. In the subtropical Gulf of Mexico (GoM), the life cycle of ABT begins with adults spawning offshore from April to June (Stokesbury *et al.*, 2004). Females produce >10 million eggs (Aranda *et al.*, 2013) and individuals hatch in 1–2 days (Tanaka *et al.*, 2014). Within 2–4 days, larvae begin exogenous feeding at a size of ~3 mm length (Malca *et al.*, 2017) and weigh ~0.1 mg DW (dry weight) (Laiz-Carrión *et al.*, 2015). The pelagic larval duration lasts 3–4 weeks (Fukuda *et al.*, 2014), during which time individuals grow quickly (~0.4–0.7 mm d^−1^) (Muhling *et al.*, 2017; Malca *et al.*, 2017). Upon yolk sack absorption, larvae depend entirely on zooplankton (e.g. ciliates and copepod nauplii) ranging in size from ~100 to 400 μm to meet their metabolic requirements (Llopiz *et al.*, 2015; Tilley *et al.*, 2016; Shiroza *et al.,* this issue). Soon after, larvae begin feeding primarily on mesozooplankton and become increasingly piscivorous at 6–8 mm (Llopiz and Hobday, 2015; Llopiz *et al.*, 2015; Uriarte *et al.*, 2019).

The extensive research on ABT early life stages provides a unique opportunity for development of IBMs. In addition, the relationships between lower trophic level dynamics and larval ecology in the GoM has recently been documented in detail as part of the Bluefin Larvae in Oligotrophic Ocean Foodwebs: Investigating Nutrients to Zooplankton in the Gulf of Mexico (BLOOFINZ-GoM) project (Gerard *et al.,* this issue). In this study, we utilize this recent work to guide the development of a spatially explicit, Lagrangian, IBM that simulates dispersal, growth and mortality with an emphasis on the period from egg to postflexion. To our knowledge, the model is the first of its kind for ABT in that it incorporates realistic spatiotemporally varying predator and prey fields that also evolve with simulated larvae through ontogeny. The modeling framework implemented here provides an important next step toward recruitment forecasting within an ecosystem based management approach. The goals for this study were to: (i) estimate annual larval mortality; (ii) compare the relative magnitudes of predation and starvation; and (iii) identify regions in the GoM that minimize larval mortality.

## METHODS

### Biogeochemical model description

The BLOOFINZ-Individual Based Model (BLOOFINZ-IBM) developed here is forced with 20 years (1993–2012) of realistic hydrodynamics, zooplankton biomass, temperature, water clarity and ambient light fields obtained from the 3D biogoechemical model NEMURO-GoM (Shropshire *et al.*, 2020). NEMURO-GoM is a highly modified version of the NEMURO biogeochemical model (North Pacific Ecosystem Model for Understanding Regional Oceanography, Kishi *et al.*, 2007) run in an offline configuration of the MITgcm (Massachusetts Institute of Technology general circulation model, Marshall *et al.*, 1997; McKinley *et al.*, 2004) and forced with dynamical fields from a ~4-km horizontal resolution, data-assimilative HYCOM (Hybrid Coordinate Ocean Model, Chassignet *et al.*, 2009; Metzger *et al.*, 2014) simulation of the GoM. NEMURO-GoM has the same horizontal resolution as the dynamical fields and includes 29 depth-constant levels (10 m intervals from 0 m to 150 m and variable resolution deeper). For more information on the numerical configuration of NEMURO-GoM see Shropshire *et al.* (2020).

NEMURO-GoM was developed specifically to examine regional zooplankton dynamics in the GoM and has been extensively validated against a combination of remote and *in situ* measurements including: mesozooplankton biomass and grazing rates, microzooplankton grazing rates, phytoplankton growth rates, net primary production, surface chlorophyll and vertical profiles of chlorophyll and nitrate (Shropshire *et al.*, 2020). The model has 11 state variables: six nonliving pools, two phytoplankton and three zooplankton functional groups. We briefly describe the latter, which is used to estimate predator and prey fields for simulated larvae. The zooplankton community is composed of small zooplankton (SZ), which represents heterotrophic protists (e.g. ciliates). Metazoan zooplankton are modeled as large zooplankton (LZ) that represent suspension-feeders and larger predatory mesozooplankton (PZ). Together, these state variables are used to approximate zooplankton biomass in three size classes 0.002–0.2 mm (SZ), 0.2–1.0 mm (LZ) and 1.0–5.0 mm (PZ).

### Lagrangian model description

Lagrangian simulations were performed using the MITgcm floats package (Adcroft *et al.*, 2018) which incorporates a fourth order Runge–Kutta scheme and is run in parallel with NEMURO-GoM. To simulate realistic spawning, particles (i.e. eggs) were released daily from 1 April to 30 June from 1993 to 2012. The number of eggs released in each grid cell were scaled proportionally to the Domingues *et al.* (2016) ABT habitat index. This index is designed to identify regions (>200 m isobath) in the GoM where larvae are likely to be found as a function of sea surface temperature and height, and geostrophic velocity. In total, 750 875 neutrally buoyant, passive particles were initialized (mean = 413 d^−1^) during the spawning period where initial depths were set randomly within the mixed layer (5–35 m). Each particle represents a “super individual” (i.e. a group of 1000 physiologically identical individuals that experience identical environmental forcing) (Scheffer *et al.*, 1995). Three-dimensional fields of zooplankton biomass, temperature, water clarity and ambient light are interpolated to particle positions every 6 hours. These particle attributes are then used to simulate growth and mortality of ABT early life stages in the IBM.

For larval ABT, the onset of piscivory is closely timed with the transition from flexion to postflexion stage, occuring around 2 weeks post hatch (Blanco *et al.*, 2019; Laiz-Carrión *et al.*, 2019; Llopiz and Hobday, 2015; Uriarte *et al.*, 2019). Larvae in the model are tracked for 3 weeks, fully encompassing the period when they are obligate planktivores. We focus our analysis on the period prior to piscivory because NEMURO-GoM does not explicitly simulate larger motile prey. Although the IBM does not simulate preflexion, flexion and postflexion stages directly, we utilize measured weights from larvae collected in the field as a reference for determining developmental stage of simulated larvae. Based on larvae collected in the GoM, postflexion occurs at ~6 mm (Shiroza *et al.,* this issue) which corresponds to ~10 days post hatch (dph) and 0.54 mg DW based on relationship presented in Malca *et al.* (2017) and Laiz-Carrión *et al.* (2015) (see supplemental S4). Thus, in our model simulated larvae weighing <0.54 mg DW are considered to be obligate planktivores.

### IBM description

The BLOOFINZ-IBM includes three life stages (eggs, yolk-sac and feeding larvae). Egg stage duration (hours (h)) is determined from an empirical temperature relationship (h = 4.66·exp(−0.11·θ)), where θ is temperature (Gordoa and Carreras, 2014). ABT eggs develop quickly in the warm water of the GoM and hatch within 48 hours. The probability that an egg will hatch is estimated using a temperature relationship presented in Reglero *et al.* (2018). The maximum probability of hatching (72%) occurs at 25°C; eggs that experience temperature <18°C or >33°C will not hatch. Upon hatching, particles are classified as yolk-sac larvae, and their lengths are prescribed based on a length-to-age relationship (Malca *et al.*, 2017). In the model, growth in weight is dynamic (see eq 1) whereas length increases monotonically with age as a function of temperature with a Q_10_ = 2. That is, growth in length increase exponentially with temperature such that every *+*10°C results in a *+*2-fold change.

Although no function exists for the rate of yolk utilization, exogenous feeding is known to begin within 2–4 dph (Tanaka *et al.*, 2014). Here, we assume exogenous feeding begins on average at 2.0 dph to be consistent with otolith-based aging studies in the region (Malca *et al.*, 2017). The influence of temperature on the yolk-sac stage duration is again included using Q_10_ = 2.0. Once exogenous feeding begins, larvae are assumed to have utilized all egg yolk (i.e. there is no overlap between endogenous and exogenous feeding). Post yolk-sac larval weights are initialized based on a weight-to-age relationship determined from larvae collected in the GoM (Malca *et al.*, 2017; Laiz-Carrión *et al.*, 2015).

Finally, to simulate feeding larvae, a bioenergetics model was developed where growth in mass occurs if the assimilated fraction (α) of total ingestion (I_tot_) exceeds the metabolic requirement (R). Larval weight (W) is updated every 6 hours using (eq 1). Starvation- and predation-induced mortality is estimated while larvae grow and are advected through the GoM as determined by ingestion, metabolism, starvation, and predation submodules described below.

(1) \begin{equation*} {\mathrm{W}}_{\mathrm{t}+1}={\mathrm{W}}_{\mathrm{t}}+\left({\mathrm{I}}_{\mathrm{t}\mathrm{ot}}\bullet \alpha -\mathrm{R}\right) \end{equation*}

### Ingestion module

Clearance rate (m^3^ larva^−1^ d^−1^) is modeled as a function of the 2D field of view fraction (φ), sensory radius (s_p_) when feeding on zooplankton prey (i.e. p = SZ, LZ, PZ), fraction of time spent feeding in a day (Δt), and the average swimming speed of larvae (v). Clearance rate is then multiplied by prey biomass (p (i,j,k,t)) at the simulated larvae’s instantaneous local position and time to estimate encounter rate (mg C d^−1^). The product of the encounter rate and capture success (σ_p_) gives ingestion rate (I_p_, mg C d^−1^):

(2) \begin{equation*} {\mathrm{I}}_{\mathrm{p}}=\varphi \pi{\mathrm{s}}_{\mathrm{p}}^2\bullet \mathrm{v}\bullet \Delta \mathrm{t}\bullet \mathrm{p}\left(\mathrm{i},\mathrm{j},\mathrm{k},\mathrm{t}\right)\bullet{\sigma}_{\mathrm{p}} \end{equation*}

Field of view in larval fish is determined by the orientation of eyes on the head. Here, we assume that larvae do not perceive prey below their horizontal plane (φ = 0.5). A swimming speed of two body lengths s^−1^ is used for all larvae, which is approximately the average cruise swimming speed observed for cultured larval tuna (Sabate *et al.*, 2010). Larvae are visual feeders (Llopiz and Hobday, 2015) and hence simulated feeding is restricted to daylight hours. The number of feeding hours in a day is estimated using an analytical function of latitude and day of the year. Prey biomass is derived from zooplankton biomass fields estimated by NEMURO-GoM. Unlike other pelagic larval fish, such as mackerel, which have highly variable prey capture success through ontogeny (Hunter, 1972), the capture success for larval tuna in rearing experiments is high (>70%), even at first-feeding (P. Reglero, unpub.). This is likely due to their large-mouth size relative to prey (Shiroza *et al.,* this issue) and hence the capture success is assumed to be constant (80%). An upper limit for ingestion is set using a temperature-dependent gut turnover time (3 h at 26°C (Young and Davis, 1990), with a Q_10_ = 2.0) and a full gut size equivalent to 10% body mass.

Ingestion is most sensitive to visual sensory radius because it is a squared term. Hence, many mathematical formulations of sensory radius have been determined from laboratory feeding studies (Hunter, 1972), by examining the anatomy of the eye (Hilder *et al.*, 2019), or derived theoretically (Aksnes and Giske, 1993). To estimate sensory radius, we utilize a recently determined anatomical relationship for the visual acuity of larval tuna (Hilder *et al.*, 2019) along with a theoretical model of visual predation derived by Aksnes and Utne (1997) to account for the impact of light and water clarity. This formulation computes sensory radius as a function of larval length, prey size, water clarity and ambient light (see online Appendix 1). Because the IBM includes many parameters (see Supplementary Table S1) we conducted a parameter sensitivity experiment using a simple individual parameter perturbation where each parameter was varied by *±*10% to investigate the impact on survival to postflexion (Supplementary Fig. S1) and postflexion age (Supplementary Fig. S2).

### Prey field module

**Fig. 1:**
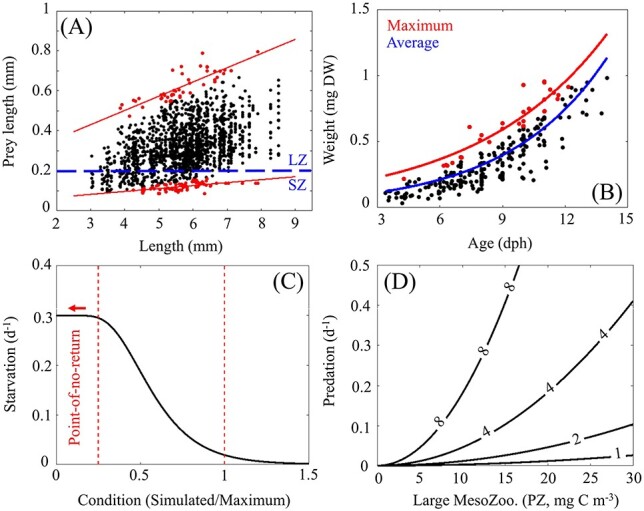
(A) Relationship between larval length (mm) and prey length (mm) from gut content analysis of 255 individuals collected in the GoM (Shiroza *et al.*, this issue). Upper and lower bounds of prey size are shown in red. Blue dotted line defines the break between small zooplankton (SZ, 0.02–0.2 mm) and large zooplankton (LZ, 0.2–1 mm) NEMURO-GoM state variables. (B) Relationship between larval weight (mg DW) and age (days post hatch) for individuals collected in the GoM. (C) Starvation as a function of an individual’s condition where >1.0 indicates ideal condition. Condition below 0.25 is used as a threshold for the “point-of-no-return” where larvae experience irreversible starvation (increased to 1.0 d^−1^ (not shown)). (D) Predation on egg and larvae as a function of simulated large mesozooplankton (e.g. PZ, 1–5 mm) biomass and example curves of individual length at 1, 2, 4 and 8 mm.

For larval ABT, prey size range changes rapidly through ontogeny. This shift is parameterized in the model based on gut content measurements from larvae collected during BLOOFINZ-GoM cruises (Shiroza *et al.,* this issue). Specifically, we determine upper and lower bounds of prey size as a function of larval length and use this to calculate the fraction of SZ (0.02–0.2 mm), LZ (0.2–1 mm) and PZ (1–5 mm) biomass that is available to simulated larvae as they grow (Fig. 1A). We note that density-dependent factors are not included in our model and hence larvae do not feed on one another or reduce zooplankton biomass in a given grid cell. The biomass of LZ and PZ are assumed to be evenly distributed over their respective size ranges which is supported by mesozooplankton biomass measurements in the region (Landry and Swalethorp, this issue). By contrast, SZ biomass is assumed to follow a size spectra relationship with a slope of zero (i.e. biomass within 0.002–0.02 mm is equal to biomass within 0.02–0.2 mm).

### Metabolic requirement module

Metabolic requirement (R, mg C d^−1^) is estimated from a weight-to-age relationship based on larvae collected in the GoM (Malca *et al.*, 2017; Laiz-Carrión *et al.*, 2015) (Fig. 1B). The derivative of this relationship gives average growth rate in mass (dW/dA). To convert to carbon, the growth rate is multiplied by a carbon to dry weight ratio (c_f_ = 0.4; (Omori, 1969)). The ingestion required to meet the observed growth rate can then be estimated by dividing by the approximate gross growth efficiency of larvae (∈ = 0.3; (Houde, 1989)). Multiplying by the difference between the approximate absorption efficiency (α = 0.7; (Houde, 1989)) and ∈ gives an estimate of metabolic requirement. Finally, the impact of temperature on metabolic requirement is included using Q_10_ = 2.0, yielding (eq 3) where t_c_ is the temperature coefficient, θ(i,j,k,t) is water temperature at an instantaneous local position and time, and θ_avg_ represents the average water temperature that field-collected larvae experience prior to being collected (assumed to be 26°C).

(3) \begin{equation*} \mathrm{R}=\frac{\mathrm{dW}}{\mathrm{dA}}\bullet{\mathrm{c}}_{\mathrm{f}}\bullet \frac{\alpha -\epsilon}{\epsilon}\bullet{\mathrm{e}}^{{\mathrm{t}}_{\mathrm{c}}\left(\theta \left(\mathrm{i},\mathrm{j},\mathrm{k},\mathrm{t}\right)-{\theta}_{\mathrm{avg}}\right)} \end{equation*}

### Starvation module

To determine the probability of starvation for simulated larvae, we first identify a maximum weight at age, defined as an exponential fit to the field-collected larvae in the upper quartile of the weight–age relationship (Fig. 1B). The actual weight: maximum-weight ratio is used as a metric of larval condition. We then fit a probability distribution function to the condition values for field-collected larvae and use the associated cumulative distribution function (CDF) to determine the probability of a larvae having a given condition value or lower. Finally, we perform a reflection of the CDF (i.e. 1-CDF so that low probability results in high mortality) and scale the CDF by a maximum starvation rate parameter (0.3 d^−1^), which yields a sigmoidal function that provides a rate of mortality due to starvation given the condition (simulated weight: maximum weight) of a simulated larva (Fig. 1C). Simulated larvae experience elevated starvation of 1.0 d^−1^ if their weight falls below 25% of the maximum weight to account for irreversible starvation (i.e. “point-of-no-return”, (Yin and Blaxter, 1987)). The number of individuals in a particle (i.e. super individual) is stepped forward in time using the generalized form *N*(*t* + 1) = N(*t*) − *dt* [*m*·*N*(*t*)], where *N* is the number of larvae, *dt* is the time step, and *m* is the mortality rate (d^−1^).

### Predation module

In NEMURO-GoM, mortality on PZ (M_PZ_, mg C m^−3^ d^−1^) is modeled as a function of PZ biomass with a quadratic formulation (M_PZ_ = [PZ]^2^·m(θ), where m is a mortality parameter scaled by a function of temperature). This formulation is commonly used in biogeochemical models as a closure term to represent implicit loss on the highest trophic level due to an unmodeled predator that covaries in abundance with its prey. Hence, M_PZ_ can be treated as an approximate predation rate for large mesozooplankton. We note that both PZ biomass and grazing rates in NEMURO-GoM have been validated with field measurements which provides some confidence in the M_PZ_ rates (Shropshire *et al.*, 2020). Next, to estimate predation on ABT early life stages, we assume that predators feeding on ABT stages prior to postflexion (~1–6 mm) are broadly similar to predators feeding on PZ (defined as 1–5 mm mesozooplankton) given their overlap in size. With this assumption, predation can be estimated for eggs or larvae by scaling M_PZ_ by a function of size because detection distance, and hence prey capture, is limited by prey size (herein M_PZ_ is normalized by PZ biomass to give a specific predation rate, (d^−1^)). The scaling function used in our predation formulation is derived in online Appendix 1 and simplifies to M_LT_ = M_PZ_ (i,j,k,t) · (L_LT_/L_PZ_)^2^, where M_LT_ (d^−1^) is the predation rate on larvae and L_LT_ and L_PZ_ are larval length (mm) and PZ length (mm), respectively (Fig. 1D). For predation on eggs L_LT_ represents egg diameter. It is important to note that our predation formulation further assumes that (i) predator size increases with larval size such that escape and capture response increase proportionally, (ii) the predator community is dominated by visual predators and (iii) the predator community composition does not change as larvae grow.

## RESULTS

### Validation of the IBM

**Fig. 2:**
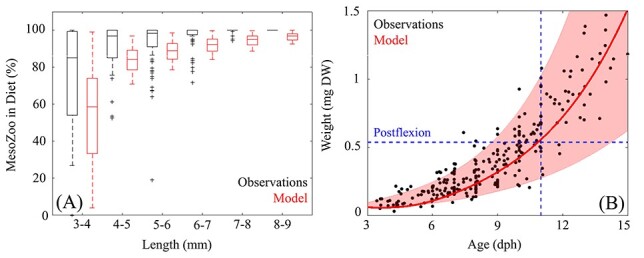
(A) Comparisons of mesozooplankton dietary contribution (% of total diet) as a function of larval length (mm) between field-collected (black) and simulated larvae (red). Whiskers extend to the 95% confidence interval. Outliers are denoted by (+) for observations and outliers for model are not shown. (B) Comparison of larval weight (mg DW) as a function of age (days post hatch) between field-collected larvae (black dots) and simulated larvae. Red line denotes model average with the 95% CI represented by shaded area. Dashed blue line denotes the average age simulated larvae reach postflexion.

The BLOOFINZ-IBM was first validated by investigating larval dietary composition (Fig. 2A). In both the guts of field-collected and simulated larvae, mesozooplankton (>200 μm) constitute the majority of larval diet. Model and field measurements align with previous studies showing high dietary contributions from mesozooplankton (Young and Davis, 1990; Llopiz and Hobday, 2015; Tilley *et al.*, 2016). Across larval size classes, mesozooplankton contributed 27–100% (95% CI) in field-collected larvae and 4–100% in simulated larvae. The majority of variability occurs in first-feeding larvae (3–4 mm size class), where mesozooplankton contributed 27–100% (median = 85%) for field-collected larvae and 4–99% (median = 59%) for simulated larvae. Dietary contribution in the 4–9 mm size class varied from 76% to 100% (median = 100%) for field-collected larvae and 66–100% (median = 85%) for simulated larvae. We note that while larvae are known to become increasingly piscivorous after postflexion, only five instances of piscivory were identified in the guts of 75 postflexion larvae (5.1–8.5 mm) collected during the BLOOFINZ cruises (Shiroza *et al.,* this issue) providing further confidence in model simulated prey fields prior to postflexion.

Larval weights simulated by BLOOFINZ-IBM also closely match observations with a correlation of 0.94 (*P* < 0.01) (Fig. 2B). On average, field-collected larvae reached postflexion weight at 10.33 dph (Malca *et al.*, 2017; Laiz-Carrión *et al.*, 2015) while simulated larvae were 10.37 dph. Herein 10 dph is referenced as “early postflexion” larvae and 3 dph is referenced as “first-feeding” larvae. The age of postflexion for simulated larvae varied from 8.5 dph to 14.5 (95% CI) dph. Larvae advected on the shelf reached postflexion more quickly because of the abundant food. In contrast, larvae reached this stage much later in the highly oligotrophic regions of the GoM. Prior to postflexion, field-collected larvae weigh 0.24 *±* 0.13 mg DW whereas simulated larvae weigh 0.27 *±* 0.13 mg DW. Although our model is expected to become more inaccurate as individuals move toward an increasingly piscivorous diet, we find nearly identical agreement between weights of simulated and field-collected postflexion larvae. On average, field-collected postflexion larvae weigh 1.03 *±* 0.59 mg DW whereas simulated larvae evaluated at the same age weigh 1.04 *±* 0.60 mg DW.

### Temporal variability in larval survival

**Fig. 3:**
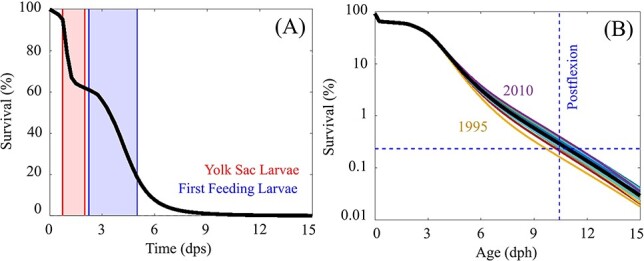
(A) Model estimated survival (%) as a function of time (days post spawn) with red shaded area denoting yolk-sac larvae and blue shaded area denoting the period when individuals begin exogenous feeding. (B) Model estimated survival (%) as a function of age (days post hatch) for each simulated spawning season (1993–2013). Black line represents mean of all years.

During the first week after spawning, the model predicts two significant mortality events (Fig. 3A). The first event involves hatching success. Eggs hatch in 18–48 hours (mean = 26 hours), with >28% of eggs never hatching and hence survival declines rapidly within the first 2 days post-spawning. Mortality slows briefly once individuals become yolk-sac larvae, with only marginally higher predation relative to eggs. Exogenous feeding begins on average at 2.12 dph and within 24 hours the model predicts a second mortality event associated with a distinct critical period lasting ~3 days (3–6 dph). During this time, survival decreases by an order of magnitude. Across all 20-years of the simulation, survival to postflexion averaged 0.24 *±* 0.05% and varied from 0.12% to 0.32% (Fig. 3B). This result suggests that recruitment in the western ABT stock could vary by a factor of 2.7 due to interannual variability in early life stage mortality alone.

In terms of model sensitivity, we find that survival to postflexion was most sensitivity to the parameters: HatchProb_p1–3 (hatching probability coefficients), ϵ (gross growth efficiency), GutTurn (gut turnover time), and GutFull (gut fullness) (Supplementary Fig. S1). Similarly, the age when larvae reached postflexion was also most sensitive to these parameters as well as FieldSampleTmpAvg (average water temperature of collected larvae) and Age2Length_p1 (slope of the growth in length curve) (Supplementary Fig. S2).

### Sources of larval mortality

**Fig. 4:**
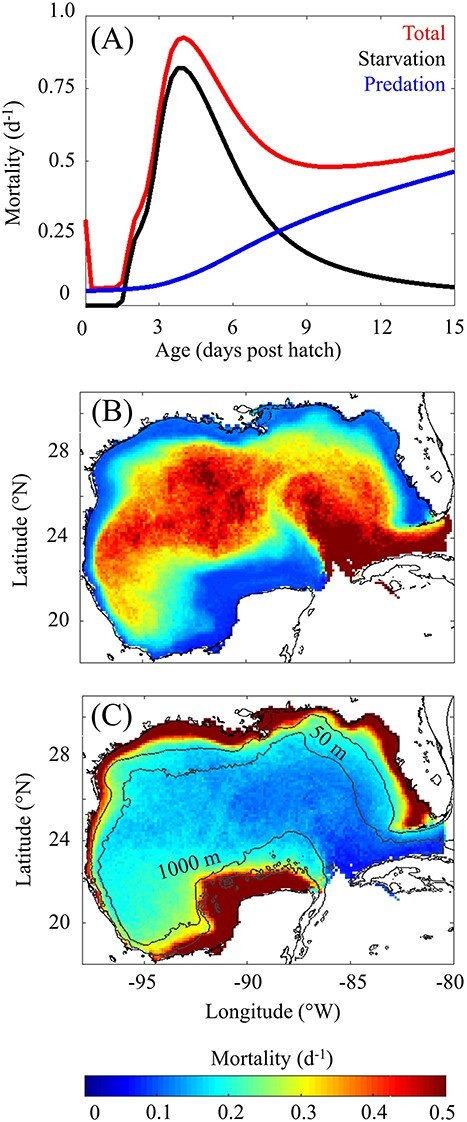
(A) Mortality rate (d^−1^) a function of age (days post hatch) with total (red), starvation (black) and predation (blue) plotted separately. (B) Spatial variability of average starvation (d^−1^) prior to postflexion. (C) Spatial variability of predation (d^−1^) prior to postflexion. Averages in starvation and predation maps are computed by organizing particles within 0.12° × 0.12° spatial bins. Black lines denote the shelf break region defined between the 50 m and 1000 m isobath.

Our analysis reveals that starvation is the largest cumulative source of mortality prior to postflexion (Fig. 4A), accounting for 49% of all larvae followed by hatching success (29%) and predation (20%). An additional 2% of total mortality was associated with advection out of the GoM. Across years, mortality contributions were consistent and varied by <1%. Contributions are robust even when losses are evaluated over the entire 3-week drift because of high mortality rates during the first week of life. Prior to postflexion, total mortality varied from 0.06 d^−1^ to 0.93 d^−1^ (mean = 0.53 d^−1^) which is slightly lower than the mortality rate (0.66 d^−1^) estimated by Davis *et al.* (1991). Starvation varied from 0 d^−1^ to 0.82 d^−1^ (mean = 0.35 d^−1^) whereas predation varied from 0.05 d^−1^ to 0.34 d^−1^ (mean = 0.16 d^−1^). Maximum mortality occurs at 4 dph, corresponding to the maximum rate of starvation (Fig. 4A). This result indicates that simulated larvae begin to starve <48 hours after the onset of exogenous feeding, which agrees closely with results from laboratory feeding experiments of larval tuna (Tanaka *et al.*, 2008).

To better understand why first-feeding larvae frequently starve, we investigated how prey availability evolves as larvae develop in the model. In NEMURO-GoM, SZ biomass is typically greater than LZ biomass by a factor of 3–4 in the open-ocean GoM. Hence, as larvae age and feed less on microzooplankon (SZ), they also experience a decrease in prey concentration as a result of a shift in prey size range. Prey biomass for first-feeding larvae averages 0.60 *±* 0.85 mg C m^−3^ whereas early postflexion larvae experience prey fields with 25% lower zooplankton biomass (Supplementary Fig. S3F). In addition, first-feeding larvae have lower total metabolic requirements, which averages 0.007 mg DW d^−1^ and increases by a factor of 7.5 for early postflexion larvae (Supplementary Fig. S3A). Despite these advantages, first-feeding larvae commonly starve as estimated by our model. This is a result of low clearance rates due to small sensory radii and slow swimming speeds, which aligns with previous findings from early larval fish feeding experiments (Hunter, 1972). From first-feeding to early postflexion, clearance rates of larvae increase by more than an order of magnitude (18 L d^−1^–480 L d^−1^, Supplementary Fig. S3E) leading to substantially lower starvation rates for larvae that survive the critical period. However, predation becomes an increasingly important source of mortality as larvae grow because their increased size allows predators to detect them more easily. At 7.75 dph predation becomes the largest source of mortality as estimated by our model (Fig. 4A).

### Spatial variability in starvation and predation

In the BLOOFINZ-IBM simulation, only a small fraction of particles are advected to the inner shelf over the course of their 3-week drift. To better understand the trade-off between predation and starvation on the shelf, we conducted an experiment in which the BLOOFINZ-IBM was run with random spawning throughout the domain (Supplementary Fig. S4B). That is, eggs were not initialized in proportion to the Domingues *et al.* (2016) habitat index as was done in the original simulation (Supplementary Fig. S4A). To calculate average mortality rates across the GoM, all particles in the random spawning simulation were first organized within 0.12° × 0.12° spatial bins. Because of the large difference in number of individuals across ages, particles within a bin were then organized by age and their averaged weight and mortalities computed. Finally, average mortalities from egg to postflexion were calculated by averaging over all binned ages weighing <0.54 mg DW (i.e. observed postflexion weight).

Strong spatial variability in starvation and predation is predicted by the model with elevated rates of starvation in the open-ocean GoM and elevated rates of predation on the shelf (Fig. 4B and C). In the open-ocean GoM (*>*1000 m isobath), starvation varied from 0.18 d^−1^ to 1.38 d^−1^ (95% CI, mean = 0.40 d^−1^) whereas predation varied from 0.08 d^−1^ to 0.37 d^−1^ (mean = 0.16 d^−1^). Within this region, starvation is greatest in the Loop Current and the north-western open-ocean GoM driven by low zooplankton biomass and increased temperature (Fig. 4A). By contrast, the shelf (*<*50 m isobath) supports high zooplankton biomass resulting in lower starvation rates but also supports greater abundances of predators. In this region, starvation varied from 0.07 d^−1^ to 0.26 d^−1^ (mean = 0.11 d^−1^) whereas predation varied from 0.21 d^−1^ to 1.34 d^−1^ (mean = 0.60 d^−1^).

### Spatial variability in larval survival

**Fig. 5:**
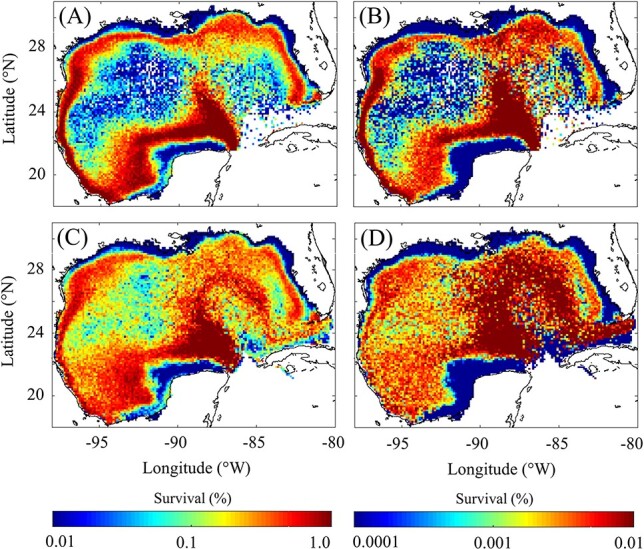
Spatial variability in survival (%) to early postflexion (A, C) and late postflexion (B, D). Survival is computed by organizing particles based on their spawning location within 0.12° × 0.12° bins (A, B) and based on their time varying location (C, D).

To investigate spatial variability in survival to postflexion, all particles from the random spawning simulation were again categorized within spatial and age bins as described in the above section (Fig. 5C). Next, the average weight and number of living individuals per particle (i.e. 0–1 000) were computed for each age. Survival to postflexion was then determined from the average number of living individuals at the binned age when larvae reached postflexion weight (i.e. first binned age with an average weight > 0.54 mg DW). Calculating survival based on average weight-at-age criteria allows one to take into account super individuals that do not reach postflexion weight. In addition, we repeated this analysis by organizing particles based on their spawning location (Fig. 5A). The first approach provides insight on how average conditions in the GoM impact survival whereas the latter provides insight on suitability of spawning locations. Both approaches reveal that the outer shelf and shelf break regions of the GoM are optimal for larval survival, minimizing the risks of starvation and predation. Based on particle spawning location, survival to postflexion varied from 0.005% to 5.78% (95% CI, mean = 0.24%) in the open-ocean GoM, <0.001–1.04% (mean = 0.20%) on the shelf, and 0.015–4.64% (mean = 0.59%) on the shelf break (*<*1000 m and *>*50 m isobaths).

To better understand the impact of predation on survival, which is the major source of mortality for older larvae, larval survival was investigated out to 7 days after postflexion (Fig. 5B, D). Although the model cannot simulate prey fields of piscivorous larvae, starvation is thought to be uncommon for late postflexion larvae, as witnessed by elevated growth rates after the initiation of piscivory (Tanaka *et al.*, 2014). Indeed, the model estimates that starvation is substantially reduced after larvae reach postflexion. This offers some confidence that the model may provide reasonable simulations until the point where larvae develop stronger swimming behavior after metamorphosis at ~25 dph (Fukuda *et al.*, 2014). For late postflexion larvae, survival decreases by more than two orders of magnitude on the shelf and varies from <0.001% to 0.01% (mean = 0.001%). Survival decreases more slowly offshore and varies from <0.001% to 0.48% (mean = 0.006%) in the open-ocean GoM, and <0.001–0.23% (mean = 0.01%) on the shelf break. This suggests that while conditions on the shelf are ideal for survival of younger larvae, survival is ultimately limited by higher rates of predation on older individuals (see Discussion).

### Habitat suitability

**Fig. 6:**
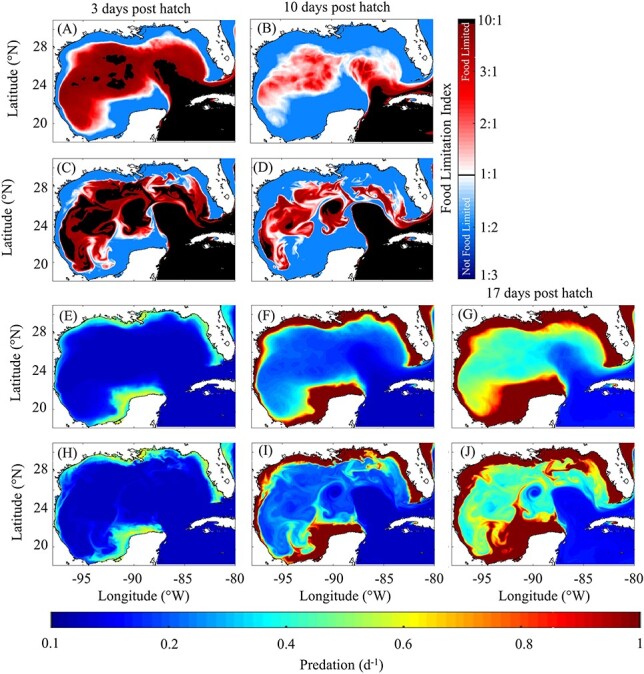
Mean and instantaneous food limitation index maps (A–D) and predation (d^−1^) maps (E–J) for the month of May. Average food limitation index map for (A) first-feeding larvae (i.e. 3 dph) and (B) early postflexion (i.e. 10 dph). Instantaneous food limitation index map on 15 May 1996 for (C) first-feeding larvae and (D) early postflexion. Average predation for (E) first-feeding larvae, (F) early postflexion larvae and (G) late postflexion larvae (i.e. 17 dph). Instantaneous predation map on 15 May 1996 for (H) first-feeding larvae, (I) early postflexion larvae and (J) late postflexion larvae.

Starvation and predation were also evaluated in an Eulerian framework to further characterize larval mortality in the GoM. Since we do not assume that past conditions influence an individual’s susceptibility to predation (i.e. the physiological condition of an individual does not impact escape response), mortality due to predation for a given age can be calculated at each grid point in the domain using the predation formulation (Fig. 6E–J). In contrast, starvation is a function of previous environmental forcing and hence cannot be evaluated in an Eulerian framework. Instead, to quantify susceptibility to starvation, we developed a food limitation index (FLI). The FLI is defined as the ratio of metabolic requirement to total assimilated ingestion (FLI = R/(I_tot_ ·α)), where values >1.0 indicate food limitation. These maps provide snapshots of whether a larva at a given age could satisfy its metabolic requirements at any time and location in the GoM (Fig. 6A–D).

Daily FLI and predation maps were computed each day over the 20-year simulation during the spawning period. Consistent with the high rates of starvation estimated by the model, we find that average prey biomass in the open-ocean GoM is insufficient to meet metabolic requirements for first-feeding larvae (Fig. 6A, C). Food limitation is so severe that metabolic requirement commonly exceeds assimilated ingestion by an order of magnitude. In terms of daily spatial extent, food limitation for first-feeding larvae varies from 82% to 98% (95% CI, mean = 95%) of the open-ocean GoM. Food limitation decreases in severity and extent for early postflexion larvae (Fig. 6B, D) varying from 26% to 88% (mean = 72%) and is typically confined to the Loop Current and GoM interior where Loop Current eddies are common (Fig. 6B). The spatial extent of food limitation increased from April to June for both first-feeding and early postflexion larvae driven by decreased prey biomass. We note that increased temperature later in the spawning period had an approximately neutral impact on food limitation because larvae grew (in length) faster, which increased their clearance rates, but also had greater metabolic requirements. On average, food limitation for first-feeding larvae covered 92% of the open-ocean GoM in April, 95% in May and 97% in June. For early postflexion larvae, food limitation has greater variability across the spawning period covering 55% of the region in April, 74% in May and 83% in June.

Predation maps show the expected inverse relationship, with elevated predation on the shelf relative to open-ocean regions (Fig. 6 E–J). In the open-ocean GoM, predation is quite consistent across the spawning period averaging 0.06 d^−1^ for first-feeding larvae and 0.20 d^−1^ for early postflexion larvae. This consistency is driven by decreased zooplankton biomass (decreasing predation) and increased temperature (increasing predation) across the spawning period which act as competing factors that largely cancel. By contrast, predation on the shelf increases across the spawning period driven by higher zooplankton biomass and temperature. For early postflexion larvae on the shelf, predation is on average 1.10 d^−1^ in April, 1.30 d^−1^ in May and 1.48 d^−1^ in June.

## DISCUSSION

ABT are highly selective spawners with adults traveling long distances from feeding grounds in the North Atlantic to spawning grounds in the GoM (Block *et al.*, 2001). Once in the GoM, adults spawn offshore over a short period of 6–8 weeks (Muhling *et al.*, 2010). This highly selective behavior suggests that there is some unique characteristic of the open-ocean GoM that favors offspring survival. Previous studies have statistically identified larval habitat within the GoM (Lindo-Atichati *et al.*, 2012; Wilson *et al.*, 2005). However, the underlying mechanisms that make the open-ocean GoM more favorable for larval survival than shelf regions, and to a larger extent, more favorable than surrounding seas has yet to be identified. Understanding the spatial variability in larval survival within the GoM is the primary objective of this study and can provide insight into the factors that create optimal spawning locations. Identifying these factors and monitoring their year-to-year variability could provide valuable information to help better predict ABT recruitment fluctuations.

### Model-data misfits

The BLOOFINZ-IBM successfully resolves key dynamics pertaining to larval ecology of ABT, including realistic larval diet and weight as a function of age, stage duration, required time for the onset of starvation, and a distinct critical period that aligns with theory (Hjort, 1914). However, some model-data discrepancies exist. The model slightly overestimates the contribution of microzooplankton to larval diet across all size classes. This discrepancy may result from poor preservation of soft-bodied microzooplankton (e.g. aloricate ciliates) in fish gut contents, leading to an underestimate in the field data. Alternately, this model-data mismatch may arise from an overestimation of SZ biomass by NEMURO-GoM or errors in the IBM ingestion formulation. Simulated larvae have strict size-thresholds for prey availability that change with age, but are otherwise not selective. However, optimal foraging theory suggests that when multiple prey types are available, larvae should preferentially feed on larger, more calorie-rich prey items (Crowder, 1985; Barnes *et al.*, 2010). Indeed, Shiroza *et al.* (this issue) found that larvae were more selective for appendicularians and podonid cladocerans when these taxa were more abundant. Further realism could be added to our ingestion formulation by incorporating optimal foraging decisions (Visser and Fiksen 2013).

Model estimates of larval weight were found to agree closely with observations, even after early postflexion, when larvae are known to become increasingly piscivorous. However, during the first few days of exogenous feeding (i.e. 3–6 dph), the model notably underestimates larval weights. On average, simulated larvae were 31% lighter relative to field-collected larvae (data: 0.13 *±* 0.05 mg DW vs model 0.09 *±* 0.01 mg DW). This discrepancy may occur because endogenous and exogenous feeding does not overlap in our model. In reality, larvae may feed exogenously while still utilizing their yolk-sac. Furthermore, processes such as microscale turbulence or prey motility that can increase encounter rates under some circumstances are not included in our model (MacKenzie *et al.*, 1994; Fiksen and MacKenzie, 2002). Such process may be particularly important for weakly swimming first-feeding larvae and could be included in future versions of BLOOFINZ-IBM.

### Mortality sources through ontogeny

Our results indicate that predation and starvation are important sources of mortality throughout the early life stages of ABT, though their magnitude and relative importance varies spatially and with larval age. Starvation was found to be the largest cumulative source of mortality accounting for nearly half of individuals spawned. This result was found to be insensitive to maximum starvation rate and point-of-no-return starvation rate parameter values. Varying these parameters by *±*50% resulted in cumulative starvation loss varying from 47% to 51%. Our model predicts that most first-feeding larvae quickly reach the point-of-no-return and that starvation is significantly reduced for those that survive the 3–6 dph critical period. As a result, total loss due to starvation is largely insensitive to maximum starvation rate. Similarly, because all larvae die after reaching the point-of-no-return, the elevated starvation rate prescribed during this period only dictates how quickly larvae die, and hence had little impact on survival to postflexion. By contrast, the threshold for determining the point-of-no-return (i.e. condition value) does have an appreciable impact on total loss due to starvation. Future field and laboratory studies aimed at quantifying this threshold are needed in order to reduce uncertainty in starvation estimates.

Although starvation contributed the greatest integrated loss, our model predicts that predation increases consistently over the pelagic larval duration becoming the largest source of mortality around 1 week post hatch. Increasing predatory risk could potentially be extrapolated out until larvae reach metamorphosis at ~22 mm (Fukuda *et al.*, 2014). In laboratory experiments, late postflexion larvae and juvenile Pacific bluefin display schooling behavior as early as 25 dph (Fukuda *et al.*, 2014; Sabate *et al.*, 2010). This indicates that predation likely remains a significant source of mortality for late postflexion larvae as suggested by our model. Estimating predation during this stage provides many challenges stemming from simulating prey fields and behavior. Although starvation is likely negligible for older larvae, simulating realistic ingestion is needed for determining stage duration and hence integrated predation loss. Indeed, faster growing cohorts have been hypothesized to experience reduced predation (Meekan *et al.*, 2006). Thus, estimating predation for older larvae will likely require prey fields and ingestion formulations that account for density-dependent factors, such as piscivory. Although challenging, accomplishing this task would be particularly relevant for fisheries management when considering the potential impacts of a warming ocean. Based on our model, increased temperature will produce a trade-off between shorter stage duration (decreased predation, particularly influencing older larvae), and increased metabolic requirements (increased starvation, particularly influencing younger larvae). Identifying the net effect of this trade-off will be key to understanding how larval survival is impacted by future climate.

### Spatial variability in larval mortality and survival

The GoM is characterized by strong biogeochemical gradients from shelf to open-ocean. Our model indicates that these cross-shelf gradients produce trade-offs between risks of predation and starvation that are important for determining survival of ABT early life stages. The greatest rates of starvation estimated by the model occur in the Loop Current and north-western open-ocean GoM. Elevated starvation rates in these regions are driven by warm temperatures (increased metabolic requirement) combined with low prey biomass. This result aligns with previous ichthyoplankton surveys that found low occurrences of larvae in the Loop Current (Muhling *et al.*, 2010). High starvation rates in the western open-ocean GoM can also be attributed to the Loop Current. Large mesoscale eddies detach aperiodically from the Loop Current every 9.5 months on average and propagate westward, transporting warm oligotrophic water into the western GoM (Sturges and Leben, 2000). Their anticyclonic circulation reduces nutrient input to the surface ocean, resulting in bottom-up limitation (Shropshire *et al.*, 2020), and explains the high rates of starvation estimated by the model in this region.

On the shelf, simulated larvae were able to largely avoid starvation. Prey was so abundant in this region that larvae quickly became satiated and hence spawning further inshore did not provide an additional growth advantage (Supplementary Fig. S5). Larvae were most successful if they were spawned near the shelf break where they experienced high prey concentrations during the 3–6 dph critical period while predation risk was still low. Individuals spawned in this region were then more likely to be advected further offshore, minimizing predation as they grew and increasing their chance of survival. Such conditions commonly occurred during the model simulation in places like the Yucatan Peninsula. Here, the Loop Current entrains plankton-rich water offshore and could explain why high abundances of larvae have been found in this region previously (Richards *et al.*, 1989). In the northern GoM, larval occurrence maps derived from historical net collections also broadly agree with the region of maximum survival predicted by the model (Muhling *et al.*, 2017). We note that when survival was evaluated for late postflexion larvae, the region of maximum survival again occurred near the shelf break, although shifted further offshore. This result suggests that elevated rates of predation on older larvae is the main factor limiting survival and may help to explain why spawning in the GoM occurs offshore.

Identifying why ABT spawn in the GoM as opposed to other nearby seas will require future comparative modeling efforts and is beyond the scope of this study. However, our model reveals that cross-shelf transport is an important factor for increasing offspring survival and may be a determining characteristic for ABT spawning grounds. In the GoM, the Loop Current, Mississippi River, and regional wind forcing create pathways for exporting large amounts of shelf water offshore (Ohlmann *et al.*, 2001; Morey *et al.*, 2013; Otis *et al.*, 2019). During its protracted phase, the Loop Current can extend far enough north to directly entrain coastal water offshore (Wiseman and Dinnel, 1988; Schiller *et al.*, 2011). More commonly, large mesoscale eddies generated by the Loop Current entrain shelf water themselves or through the counter vortices they produce. These eddies exist throughout the year and are thought to be the main mechanism driving cross-shelf transport in the region (Ohlmann *et al.*, 2001; Morey, *et al.*, 2003a; Brokaw *et al.*, 2019). By contrast, the contribution of cross-shelf transport driven by the Mississippi River and local wind forcing is highly seasonal. During fall and winter, easterly winds favor transport of riverine water along the coast inhibiting cross-shelf (Walker *et al.*, 2005; Morey, *et al.*, 2003b). However, during spring and summer, southerly winds favor Ekman transport to the east of the Mississippi Delta over deeper waters (Ohlmann *et al.*, 2001; Morey, *et al.*, 2003b). In addition, cold fronts during the winter and spring create wind reversals, which can rapidly transport coastal water offshore. The timing of these cross-shelf events may provide additional advantages for offspring survival in the GoM. Future studies should investigate the magnitude of cross-shelf transport in regional oceans adjacent to the GoM and other spawning grounds to identify the importance of this regional characteristic.

### Application to stock assessments and future work

Ocean models are well-suited for evaluating larval mortality for species like ABT because: (i) early life stages develop in pelagic waters influenced by large-scale ocean circulation (e.g. Loop Current) that can be well resolved by hydrodynamic models, (ii) their pelagic larval duration is short and (iii) their low-trophic-level food is strongly influenced by bottom-up forcing resolved by biogeochemical models. The present modeling framework could be updated routinely to give real-time and future predictions of larval survival. These estimates could potentially be used to inform future expected recruitment within stock assessment models that form the basis of catch limits for managing ABT fisheries.

Further realism could be added to BLOOFINZ-IBM framework for increasing the model’s utility in management applications. In particular, particles could be initialized based on annual ichthyoplankton survey data to provide more realistic particle release locations. Realism could be added by incorporating the impact of maternal effects such as initializing egg weights based on the condition of spawning females. Because ABT are selective feeders, even within mesozooplankton size class (Shiroza *et al.,* this issue), added realism may also be achieved by combining NEMURO-GoM with a zooplankton food web model (Stukel *et al.,* this issue) to improve ingestion estimates. We note that initial comparisons of ABT recruitment times series and yearly larval survival predicted by the model do not agree well. This could indicate that the aforementioned additions are necessary to increase the model’s predictive ability. Alternatively, this mismatch could indicate that processes during juvenile stages are also important for accurate recruitment forecasting.

One of the main advantages of the modeling framework developed here is that it lends insights into the potential factors that may cause significant changes in recruitment success of ABT over time. The ABT population has experienced regime-shift-type behavior in the past; observed recruitment declined dramatically after 1975 and it has been suggested that this is due to environmental forcing (Brown *et al.*, 2002). Uncertainty in the cause of recruitment declines greatly complicates management of the species because assumptions regarding stock productivity have major influence on reference points, and there is debate over whether the historically observed elevated recruitment levels should be expected in the future or if more recent lower recruitment levels represent a new norm (Porch and Lauretta, 2016). Understanding mechanisms for variations in recruitment within the early life stages is critical to understanding whether there is evidence for environmentally driven shifts in recruitment, or whether these shifts are the result of other population dynamics such as changes in spatial distribution and overexploitation (Fromentin *et al.*, 2014). Modeling work has also suggested that ABT will be highly impacted by climate change as a result of their narrow temperature preferences for spawning (Muhling *et al.*, 2011). The present study highlights critical bottlenecks where larvae experience high mortality, which include temperature-dependent processes, and hence can provide further insight into the potential impacts of climate change on the species.

## CONCLUSIONS

Other regions in the Atlantic and Caribbean Seas contain similar conditions to the GoM (e.g. warm oligotrophic water), yet show no evidence of large-scale spawning. Our results indicate that the GoM may provide an ideal spawning ground because of the region’s large shelf and strong mesoscale activity. Together these regional characteristics increase the chance of shelf water entrainment into highly oligotrophic regions that may be crucial for ensuring both low starvation during the critical period and low predation later in development. However, future IBM studies that compare larval mortality in nearby regional oceans are needed to conclusively identify the importance of cross-shelf transport for spawning grounds. Within the GoM, it has been hypothesized that, despite the potential abundant prey on the shelf, ABT spawn in offshore regions to minimize predation on their larvae. Our results unequivocally support this hypothesis indicating that although starvation is the largest source of mortality, higher predation rates on older larvae ultimately limits survival.

## DATA ARCHIVING

Data from field-collected larvae presented here have been submitted to the National Oceanic and Atmospheric Administration’s (NOAA) National Centers for Environmental Information (NCEI) data repository and will also be archived at BCO-DMO (Biological and Chemical Oceanography Data Management Office) site https://www.bco-dmo.org/program/819631.

## Supplementary Material

tshropshire_JPR_S2_appendix_022321_fbab041

tshropshire_JPR_S2_supplemental_022321_fbab041
