# Supplementary material for: Trade-offs between risks of predation and starvation in larvae make the shelf break an optimal spawning location for Atlantic bluefin tuna
Source: J Plankton Res. 2021 Jun 22;44(5):782–98. doi: 10.1093/plankt/fbab041 (PMC9424715; doi:10.1093/plankt/fbab041)
Supplement: tshropshire_JPR_S2_appendix_022321_fbab041 [file tshropshire_jpr_s2_appendix_022321_fbab041.docx]

**Online Appendix 1: Derivation of larval fish sensory radius and predation-induced mortality**

**Sensory radius derivation**

First, we obtain the maximum reactive distance (MRD) for larval tuna from the minimum separable angle (MSA) using the relationship MSA = 4.699*L_lt_^-1.129^ (Hilder *et al.*, 2019), where L_lt_ is larval length. The MSA takes into account the density of light sensors on the retina (e.g. cones) along with the geometry of the eye lens to determine the minimum angle which can be distinguished by a predator. The MRD for a prey item of a given size (L_p_) can then be determined using (eqA1).

$\mathrm{MR}D_{p}=\frac{0.5 \cdot L_{p}}{tan(0.5 \cdot MSA)}$ (eqA1)

The reactive distance using this method serves as a theoretical anatomical maximum. To include the impact of light availability and water clarity on the reactive distance of larval tuna we utilize a theoretical model of aquatic visual predation developed by Aksnes and Utne (1997) (eqA2). The model assumes that a predator (in this case larval tuna) can detect a prey only if the difference between the retinal irradiance flux produced by a prey image and background is greater than some threshold. Unlike the minimum separable angle approach, the Aksnes and Utne (1997) model includes the impact of prey size as well as contrast to calculate reactive distance.

The Aksnes and Utne (1997) model includes a saturating light term where I_m_ is the maximum possible irradiance the visual system of a predator can process, I_z_ is the ambient light for a given depth, and k_i_ is the light half saturation coefficient, which was determined experimentally by Aksnes and Giske (1993). The light limitation term is multiplied by the prey contrast (C_p_), attenuation of the contrast signal across the reactive distance (D_p_ (in meters)) with beam attenuation (c). A_p_ is the area of the prey image and ΔE is the sensitivity threshold of a predator for detecting differences in retinal irradiance flux between a prey image and background. For the full derivation of (eqA2) see Aksnes and Giske, (1993) and Aksnes and Utne, (1997).

$D_{P}^{2}=\frac{I_{z}\cdot I_{m}}{I_{z}+k_{i}}\cdot C_{p}e^{-cD_{p}}\cdot\frac{A_{p}}{\Delta E}$ (eqA2)

Since we are only interested in quantifying the impact of light on reactive distance, all terms which are not influenced by light availability or water clarity can be combined into a single constant X_1_. The constant can be solved for by assuming light saturated conditions (I_k_ >>k_i_) with a prescribed beam attenuation and reactive distance in clear water. Beam attenuation is related to light attenuation (k_tot_) using the equation c=4 *k_tot_ (Aksnes and Giske, 1993) and in clear water k_tot_ is assumed to equal 0.04. The reactive distance is set using the MRD calculated from (eqA1). Once X_1_ has been determined the reactive distance with varying light and water clarity is obtained by iteratively solving (eqA3) using D_p_= MRD as an initial condition.

$D_{p}= \sqrt{X_{1}\cdot\frac{I_{z}}{I_{z}+k_{i}}\cdot e^{-cD_{p}}}$ (eqA3)

Ambient light levels (I_z_) are obtained from NEMURO-GoM which is forced by daily averaged surface shortwave irradiance fields estimated by the CFSR (Climate Forecast System Reanalysis) atmospheric model (see Shropshire *et al.,* 2020 for more details). The impact of water clarity is obtained using the total light attenuation term estimated from NEMURO-GoM. Vertical light attenuation in NEMURO-GoM is modeled using Beer’s law where k_tot_ is a function of attenuation due to water as well as small and large phytoplankton biomass.

Assuming a conical field of view with a given angle one can obtain the sensory radius from the estimated reactive distance in (eqA3) with the equation S_p_ = D_p_ **·** tan (Ø **·** 0.5). The location of eyes in pelagic larval fish are often located towards the top of the head with little to no ability to see behind them resulting in a maximum visual field of view angle of approximately 180°. However, given the upward orientation of the eyes in larval tuna here we assume individuals perceive half of the possible perception field (e.g. Ø = 90°) which results in the reaction distance and sensory radius being equal (S_p_ = D_p_). As stated previously the MRD estimated in (eq3) is considered a theoretical maximum. Laboratory feeding studies have shown evidence suggesting the maximum behavioral reactive distance is approximately half of the anatomical MRD (Parker *et al.*, 2017). Hence, the reactive distance estimated in (eqA3) is first divided by a factor of 2.0 before calculating the sensory radius used to calculate ingestion with (eq2).

**Predation equation derivation**

To estimate predation we first we first assume that predation on larval ABT is only a function of larval size because larger prey can be detected from a greater distance. With this assumption we utilize the mortality rate on large mesozooplankton (PZ, 1 – 5 mm) estimated by NEMURO-GoM to approximate predation on larval tuna given the overlap in size. In NEMURO-GoM, mortality on PZ is modeled as a function of PZ biomass and temperature with a quadratic formulation which is often used in biogeochemical models to represent implicit loss terms on the highest trophic level due to an un-modeled predator that covaries in abundance with its prey. Specific mortality of PZ (M_PZ_) in NEMURO-GoM is expressed using (eqA8), where m is the mortality rate parameter (d^-1^) as a function of temperature.

$M_{\mathrm{pz}}=\frac{\left[ \mathrm{PZ} \right]^{2}\cdot m(\theta)}{[PZ]}$ (eqA8)

However, we could also write a mechanistic equation relating PZ mortality to the abundance of PZ, the abundance of an unmodeled predator (F) with a sensory radius (S_pz_) and swimming speed (v) (e.g., similar to (eq2), but for predator “F” feeding on PZ rather than larval ABT feeding on SZ and LZ). The concentration of PZ cancels and the equation can be written in terms of predator abundance and encounter rate (eq9).

$M_{\mathrm{PZ}}= [F]\cdot\varphi\pi S_{\mathrm{PZ}}^{2}\cdot v\Delta t\cdot\sigma$ (eqA9)

The same equation to (eq9) can be written for the specific mortality for larval tuna (M_LT_) where the predator now has a sensory radius (S_LT_). If we assume that the predators of PZ and larval ABT are broadly similar (a reasonable assumption, because of their overlap in size) the ratio of predation on larval tuna to PZ mortality is found to equal the ratio of their sensory radius squared. Predation on larval tuna can then be estimated using (eqA10) where M_PZ_ is spatiotemporally varying as estimated by NEMURO-GoM.

$M_{\mathrm{LT}}=M_{\mathrm{PZ}}\cdot\left( \frac{S_{\mathrm{LT}}}{S_{\mathrm{PZ}}} \right)^{2}$ (eqA10)

To determine S_LT_ and S_PZ_ we again start with the Aksnes and Utne (1997) visual predation model used to determine sensory radius for larval tuna. First, the reactive distance for a predator feeding on PZ is written as in (eq4). All terms that are not associated with reactive distance or prey attributes can be aggregated into a single constant X_2_ (eqA11). The underlying assumption is that the influence of light availability and water clarity will have the same impact on the ability of a predator to capture PZ or larval ABT.

$D_{\mathrm{PZ}}^{2}\cdot e^{cD_{\mathrm{PZ}}} =C_{\mathrm{PZ}}\cdot A_{\mathrm{PZ}}\cdot X_{2}$ (eqA11)

Similarly, (eqA11) can be written for the reactive distance of a predator feeding on larval tuna (D_LT_) using the prey contrast (C_LT_) and area (A_LT_). Taking the two equations, D_PZ_ can be written in terms of larval tuna attributes using two parameters (β, γ) that relate PZ size and contrast to larval tuna size and contrast.

$D_{\mathrm{PZ}}^{2}\cdot e^{cD_{\mathrm{PZ}}} =\beta C_{\mathrm{LT}}\cdot{\gamma A}_{\mathrm{LT}}\cdot X_{2}$ (eqA12)

The left hand side of (eq11) (with subscripts LT) can be substituted on the right hand side of (eq12) resulting in a single equation that relates the reactive distance of a predator feeding on PZ to the reactive distance of a predator feeding on larval tuna (eq13).

$D_{\mathrm{PZ}}^{2}\cdot e^{cD_{\mathrm{PZ}}} =\beta\gamma\cdot{(D}_{\mathrm{LT}}^{2}\cdot e^{cD_{\mathrm{LT}}})$ (eqA13)

Like most mesozooplankton, larval tuna are highly transparent. Here we assume that the contrast of PZ and larval ABT is equal (β = 1.0) and hence the term can be removed. The prey area scaling term can be expanded (i.e. γ = (π**·**0.5**·**L_PZ_)^2^/(π**·**0.5**·**L_LT_)^2^) results in (eq14).

$D_{\mathrm{LT}}=D_{\mathrm{PZ}}\cdot\frac{L_{\mathrm{LT}}}{L_{\mathrm{PZ}}}\cdot\sqrt{e^{c\left( D_{\mathrm{PZ}}-D_{\mathrm{LT}} \right)}}$ (eqA14)

Reactive distance can be replaced with sensory radius when assuming a 90° field of view. Next we solve for the ratio of sensory radii and manipulate the exponential term resulting in (eq15):

$\frac{S_{\mathrm{LT}}}{S_{\mathrm{PZ}}}=\frac{L_{\mathrm{LT}}}{L_{\mathrm{PZ}}}\cdot\sqrt{e^{c\cdot S_{\mathrm{PZ}}\left( 1 - \frac{S_{\mathrm{LT}}}{S_{\mathrm{PZ}}} \right)}}$ (eqA15)

The equation above can be solved iteratively by prescribing a value for S_PZ_ and assuming for an initial condition that the reactive distance of a predator feeding on PZ and larval ABT is equal (S_LT_/ S_PZ_ = 1). Here S_PZ_ is approximated by assuming that PZ are on average 2 mm, predators feeding on PZ (size = 2 mm) have an average predator to prey ratio equal to 10, and a reactive distance of 2 body lengths, resulting in S_PZ_ = 40 mm. We note that eqA15 is insensitive to the initial value used for S_PZ_ because the expression inside the exponential is on the order 1x10^-2^ to 1x10^-3^ resulting in predation on LT increasing approximately in proportion to the ratio of larval and PZ length squared (eqA16).

$M_{\mathrm{LT}}=M_{\mathrm{PZ}}\left( i,j,k,t \right)\cdot\left( \frac{L_{\mathrm{LT}}}{L_{\mathrm{PZ}}} \right)^{2}$ (eqA16)

During model tuning, predation rate was adjusted using L_PZ_= 2.0–4.0 mm for the average size of PZ. We chose a value of 2.0 mm. For predation during the egg stage L_LT_ is set to 1.0 mm while L_LT_ for yolk-sac and post yolk-sac larvae is set based on the previously described length to age relationship. During the egg and yolk-sac stage larvae experience lower rates of predation than mortality on PZ estimated by NEMURO-GoM given their smaller size. Mortality on larval ABT is equal to the mortality on PZ when larval ABT are the same size as PZ (i.e. 2 mm) and increases non-linearly after this point.
